# Phase 2 Study of Low-Dose Paclitaxel and Cisplatin in Combination With Split-Course Concomitant Twice-Daily Reirradiation in Recurrent Squamous Cell Carcinoma of the Head and Neck: Long-term Follow-up of NRG Oncology Radiation Therapy Oncology Group Protocol 9911

**DOI:** 10.1016/j.ijrobp.2025.07.1434

**Published:** 2025-07-31

**Authors:** Corey J. Langer, Jonathan Harris, Eric M. Horwitz, Merrill Kies, Voichita Bar, Stuart J. Wong, Jimmy J. Caudell, Kenneth L. Zeitzer, Sharon A. Spencer, Qiang Zhang, Sue S. Yom, Quynh-Thu Le

**Affiliations:** a Department of Hematology-Oncology, University of Pennsylvania, Philadelphia, Pennsylvania; b NRG Oncology Statistics and Data Management Center, Philadelphia, Pennsylvania; c Department of Radiation Oncology, Fox Chase Cancer Center, Philadelphia, Pennsylvania; d Thoracic/Head and Neck Medical Oncology Department, University of Texas MD Anderson Cancer Center, Houston, Texas; e Department of Radiation Oncology, Jefferson Einstein Hospital, Philadelphia, Pennsylvania; f Department of Medicine, Medical College of Wisconsin, Milwaukee, Wisconsin; g Department of Radiation Oncology, H. Lee Moffitt Cancer Center, Tampa, Florida; h Hazelrig-Salter Department of Radiation Oncology, Heersink School of Medicine University of Alabama at Birmingham, Birmingham, Alabama; i Department of Radiation Oncology, University of California San Francisco, San Francisco, California; j Department of Radiation Oncology, Stanford University, Stanford, California

## Abstract

**Purpose:**

Locoregionally recurrent squamous cell carcinoma of the head and neck and second primary tumors (SPTs) in previously irradiated fields, if not resectable, are virtually always fatal. Chemotherapy alone yields a median survival of 10 to 11 months and 5-year overall survival (OS) rates of <5%. Concurrent reirradiation and chemotherapy constitutes an alternative, nonstandard strategy. Herein, we report the long-term outcomes of NRG Oncology Radiation Therapy Oncology Group 9911, a phase 2 trial of split-course radiation therapy (RT) and concurrent paclitaxel and cisplatin.

**Methods and Materials:**

Eligibility stipulated measurable, recurrent squamous cell carcinoma of the head and neck or SPT in previously irradiated fields, performance status 0–1, and adequate end-organ indices. Patients received split course, twice-daily RT (1.5 Gy/fraction twice a day × 5 days every 2 weeks ×4), plus cisplatin (15 mg/m^2^every day × 5) and paclitaxel (20 mg/m^2^every day ×5) every 2 weeks for 4 cycles. Granulocyte colony-stimulating factor was administered on days 6 to 13 of each 2-week cycle. The primary endpoint was OS relative to historical control, NRG Oncology Radiation Therapy Oncology Group 9610. Secondary endpoints included progression-free survival, toxicities, and patterns of failure.

**Results:**

Between March 2000 and June 2003, 105 patients were enrolled; 100 patients were analyzable (76% male, median age 60 years). Oropharynx (41%) and oral cavity (27%) were the predominant primary sites. A total of 23% had SPT. Median prior RT dose was 65.7 Gy. Overall, 73% of patients completed all chemotherapy. Nine treatment-related deaths (9%) occurred: 5 in the acute and 4 in the late setting. Survival was significantly improved over historical control (*P* = .01) with 5-year survival increased from 3.8% (95% CI, 0.0–8.0) to 14.9% (95% CI, 7.9–21.9). Five-year progression-free survival was 7.0% (95% CI, 2.0–12.0). A total of 64.9% died of incident cancer, 3.2% of SPT, and 22.3% of noncancer or unknown causes. In 1-year survivors, the rate of subsequent late grade 4–5 toxicity was significantly higher than historical control (*P* = .02), with 5-year cumulative incidence of 22.4% (95% CI, 11.8–35.1) compared with 3.2% (95% CI, 0.2–14.5).

**Conclusions:**

Despite a high incidence of grade 5 toxicity, OS rates for this trial evaluating concurrent split course twice a day reirradiation with cisplatin and paclitaxel exceeded results seen historically with chemotherapy alone.

## Introduction

Locoregionally recurrent carcinoma of the upper aerodigestive tract in prior radiation fields, if not curable by surgery or additional radiation therapy (RT), is virtually always fatal. Most recurrences in the head and neck remain localized or regional, rendering them potentially amenable to additional local treatment.^1,2^ At the time Radiation Therapy Oncology Group (RTOG) 9911 was devised, cisplatin and paclitaxel, both radio-sensitizers, had demonstrated clear activity in head and neck cancer (HNC) and were major components of the standard of care.^3–6^ Uninterrupted daily RT is generally thought to be superior to split course daily RT as primary therapy, but it was possible that a split-course schedule in the relapsed setting might enable optimal integration of chemotherapy with RT. A split-course schedule also allows incorporation of hematopoietic growth factor (HGF) between cycles, avoiding the paradoxical toxicity noted with concurrent RT and HGF. HGF may also ameliorate mucositis, as well as reduce myelosuppression, the expected dose-limiting toxicities when cytotoxics are grafted onto RT. A prior phase 1 trial (Fox Chase Cancer Center, FCCC 96–006) and phase 2 trial (FCCC 99–025) demonstrated that hyperfractionated split-course RT, in combination with concurrent daily bolus cisplatin and paclitaxel, with HGF between cycles, was feasible and promising with long-term survivors.^7,8^ These various results laid the foundation for NRG Oncology RTOG 9911, a phase 2 trial in relapsed HNC integrating split course (every other week) twice-daily RT and concurrent low-dose daily cisplatin and paclitaxel. Early results were reported in the *Journal of Clinical Oncology* in 2007^9^ and were considered “promising.” At a median follow-up of 2.0 years (min-max, 0.5–3.8 years), median survival time was 12.1 months, with estimated 1- and 2-year overall survival (OS) rates of 50.2% and 25.9%, respectively.^9^ We now update the long-term survival and late sequelae of NRG Oncology RTOG 9911 with a median follow-up of 9.1 years (min-max, 5.0–10.1 years).

## Methods and Materials

### Objectives

The primary objective of this study was to determine the median and 1-year OS rates of patients with squamous cell carcinoma of the head and neck (SCCHN) treated with split-course twice-daily reirradiation with concurrent cisplatin and paclitaxel. Secondary end points included (1) median and 1-year progression-free survival (PFS), (2) evaluating the incidence of acute and late toxicities, and (3) determining the patterns of disease progression. This updated analysis evaluated long-term survival, defined as 5 years or more, as well as late toxicities.

### Patients

Eligibility stipulated pathologically confirmed, recurrent, unresectable SCCHN or second primary tumor (SPT) in a previously irradiated field, excluding nasopharynx or salivary gland tumors. The protocol did not specify criteria for defining recurrent tumor versus SPT, but allowed the investigators to use the accepted definitions at the time, which follow. SPT referred to new lesions a minimum of 2 cm from the original tumor^10^ or having the clear features of another anatomic site or a new lesion noted >5 years in the same region of the original cancer.^11^ Additional eligibility requirements required measurable tumor; >6-month interval since completion of prior chemotherapy and RT; ≥75% of the tumor volume having received prior RT at doses between 45 and 75 Gy; Eastern Cooperative Oncology Group performance status (PS) 0 to 1; and the absence of significant comorbidities. The protocol mandated that the entire tumor volume be included in a treatment field without exceeding a lifetime cumulative spinal cord dose of 50 Gy. Adequate physiological indices were required, including absolute neutrophil count ≥1,500/mL, platelets ≥100,000/mL, bilirubin ≤1.5 mg/dL, and creatinine ≤1.5 mg/dL. All patients were required to have liver function tests no >2 times upper limits of normal. Ultrasound or computed tomography (CT) of the liver showing no metastasis was required if liver function tests proved >2 times upper limits of normal. CT of the chest was mandated. All patients signed the informed consent form in accordance with institutional standards.

Exclusion criteria included distant metastases, primary tumor in the nasopharynx or salivary gland, other invasive malignancies within the preceding 2 years, pre-existing grade 2 or worse peripheral sensory neuropathy, or ongoing lactation or gestation.

### Treatment

Patients received RT at a dose of 1.5 Gy/fraction twice a day for 5 days every other week for 4 cycles; the daily inter-fraction interval was 4 to 6 hours. Chemotherapy consisted of cisplatin 15 mg/m^2^ infused over 1 hour and paclitaxel 20 mg/m^2^ over 1 hour, each given daily for 5 days, every other week for 4 cycles (8 weeks total). Standard premedication included antihistamines, corticosteroids, H2 blockers, and serotonin antagonists. Granulocyte colony-stimulating factor, either 300 mg subcutaneously daily (patients ≤60 kg), or 480 mg daily (patients ≥60 kg), was administered days 6 through 13 every other week. Intravenous fluid was administered before and during platinum infusion with appropriate electrolyte replacement.

Treatment fields encompassed gross tumor with adequate margins (≤2 cm) whenever possible. Margins of <2 cm were considered acceptable only in instances of spinal cord encroachment. Elective treatment to regional lymph nodes was not required. Individualized treatment planning with CT or conventional simulation was mandated. Positron emission tomography or positron emission tomography/CT imaging was not used for treatment planning. Review of the previous RT records (including simulation films, treatment plans, and dosimetry data) was required to avoid total spinal cord radiation in excess of 50 Gy. All fields were treated daily; custom blocking with multileaf collimation or Cerrobend (Cerro Metal Products) was used with each beam to limit radiation to the surrounding normal tissue. Conventional 3-dimensional conformal RT and intensity modulated RT (IMRT) techniques were used at the discretion of the individual investigators. For 3-dimensional conformal RT and IMRT treatment plans, iso-dose calculations in the axial, sagittal, or coronal planes were required. In addition, dose-volume histograms for the gross tumor volume and the spinal cord were required.

The doses of cisplatin and paclitaxel were reduced 25% during the second and subsequent cycles for any of the following clinical scenarios: neutropenic fever, delay in resuming radiation >1 week, thrombocytopenia requiring platelet transfusion, grade 4 mucositis requiring total parenteral nutrition or hospitalization, and grade 3 or worse fatigue, which, in the opinion of the treating physician, precluded full-dose therapy. In addition, the dose(s) of the implicated agents were reduced 25% for grade 3 neurotoxicity or other attributable grade 4 nonhematologic toxicity, excluding nausea and vomiting. Persistent creatinine elevations of 1.6 to 2.0 mg/dL mandated a 50% reduction in cisplatin dose. Cisplatin was withheld for creatinine elevations exceeding 2 mg/dL. Treatment was delayed by 1 week or more for mucositis precluding hydration or intake; grade 2 or worse neurotoxicity, absolute neutrophil count <1500/mL, or platelets <80,000/mL; or other grade 3 or worse nonhematologic toxicities.

### Statistical considerations

Using the method of Dixon and Simon,^12^ a sample size of 90 patients followed for ≥12 months ensured 80% power to detect a 15% absolute improvement in the 1-year OS rate (from 35% to 50%) compared with historical control data from NRG Oncology RTOG 9610 with a 1-sided log-rank test^13^ at the 0.05 significance level. The sample size was adjusted by 10% to allow for ineligible patients, to a total of 100. Secondary endpoints were PFS, toxicity, and patterns of failure. Failure for PFS was defined as local, regional, or distant progression, new primary tumor, or death. Failure for OS was defined as death due to any cause. OS and PFS rates were estimated by the Kaplan-Meier method.^14^ Hazard ratios were estimated by Cox proportional hazards models. The treatment effect for OS (RTOG 9911/RTOG 9610) was calculated without and with adjustment for other prognostic factors. We considered the following variables for inclusion in multivariable models: age (continuous), sex (male vs female), Zubrod PS (1 vs 0), feeding tube use (yes vs no), disease type (second primary vs recurrence), months from prior RT (continuous), prior chemotherapy (yes vs no), and prior surgery (yes vs no). The rates of late toxicity (per RTOG/European Organization for Research and Treatment of Cancer criteria)^15^ were estimated by the cumulative incidence method,^16^ with death not due to toxicity as a competing risk and compared with historical control by Gray’s test.^17^ Sample size calculations and analysis were performed in SAS version 9.2.

## Results

One hundred five patients were enrolled at 36 separate institutions between March 2000 and June 2003. Five patients proved ineligible: in 4, <6 months had elapsed from completion of prior RT to recurrence, and 1 patient died before starting treatment. One hundred patients were deemed eligible and started protocol therapy.

### Baseline demographics

Baseline patient demographics are shown in Table 1. Of the 100 analyzed patients, median age was 60 years (IQR, 51–67 years). Seventy-six percent were male, 90% White, and 66% had Eastern Cooperative Oncology Group PS 1; 23% had SPTs, and 77% had locoregional recurrences. The predominant site of recurrence was oropharynx (41%); oral cavity was the second most common site (27%). Median time from prior radiation to study entry was 39.6 months. Fifty-two percent relapsed >3 years after prior RT. The median prior RT dose was 65.7 Gy (range, 45–75 Gy). Twenty percent had received prior chemotherapy, mostly cisplatin-containing regimens.

### Acute toxicity

Toxicities per Common Toxicity Criteria category and term are shown in Table E1. Five percent (5/100) experienced early grade 5 toxicities within 3 months of treatment completion. Causes included neutropenic sepsis in 2 individuals, with death occurring 9 and 23 days from the start of treatment, dehydration and shock (71 days), pneumonitis (20 days), and cerebrovascular accident (55 days). Twenty-one percent (21/100) experienced grade ≥4 myelosuppression, including 18% (18/100) with grade ≥4 neutropenia. Twenty-one percent (21/100) experienced grade ≥3 anemia, and 6% (6/100) grade ≥3 thrombocytopenia. Eleven percent (11/100) required platelet or red cell transfusions. Grade ≥3 infection or febrile neutropenia occurred in 15% (15/100). Grade ≥3 mucositis/stomatitis occurred in only 14% (14/100); 2% (2/100) were grade 4. Fifty-one percent (51/100) had grade ≥3 GI toxicity.

### Late toxicity

**Fig. 1.**
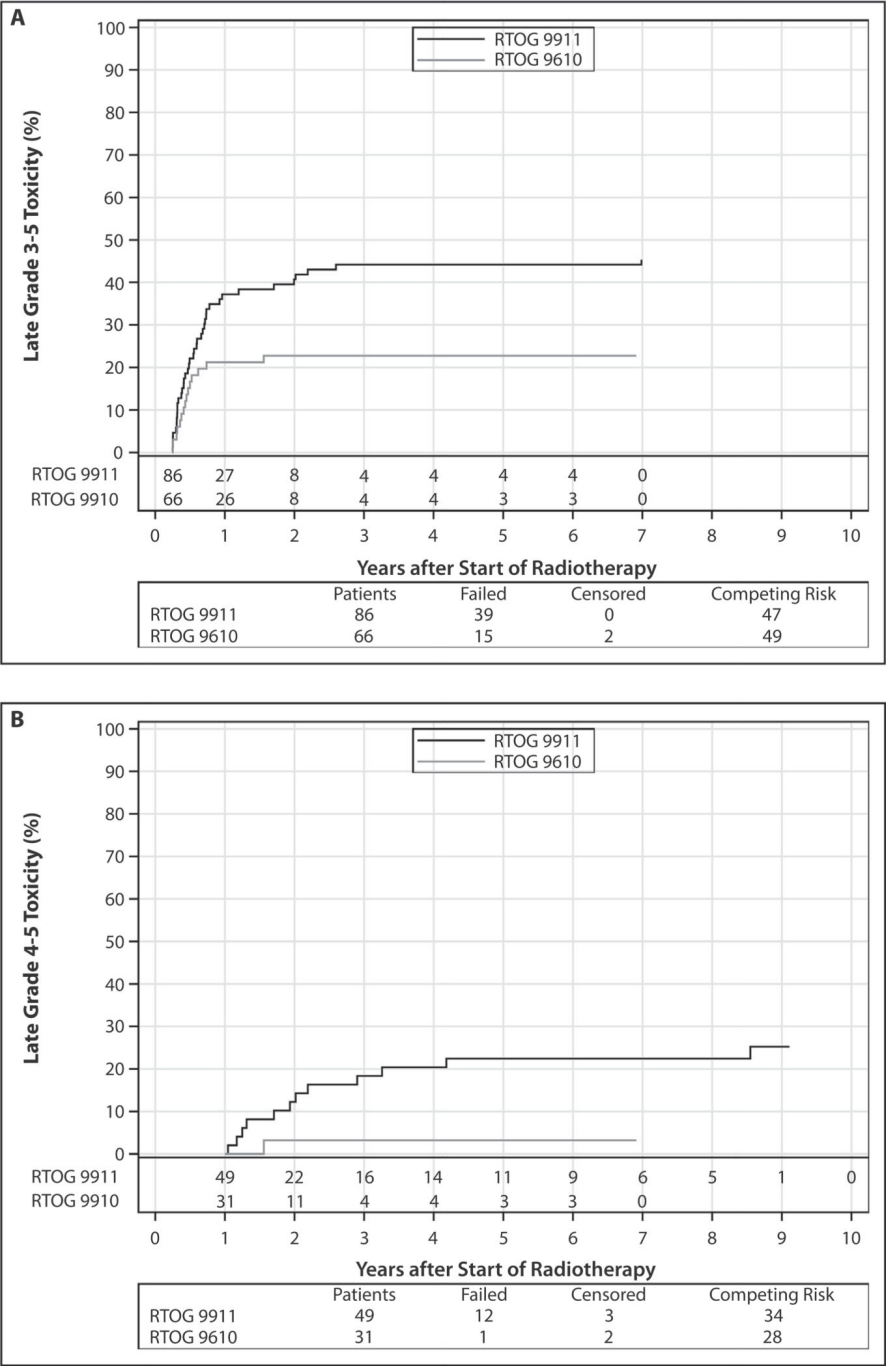
Cumulative incidence of late grade 3–5 toxicity in all patients (A) and in 1-year survivors (B) compared with historical control, Radiation Therapy Oncology Group (RTOG) 9610.

Eighty-six patients were evaluable for late toxicity (Table 2). Late grade 5 toxicities included 3 (3.5%) carotid hemorrhages at 272, 427, and 2772 days from the start of treatment, and 1 (1.2%) death attributable to oral-cutaneous fistula and soft tissue necrosis (116 days). The cumulative incidence of late grade 3–5 toxicity (using death as a competing event) was significantly higher than historical control (*P* = .01) with 5-year estimates of 44.2% (95% CI, 33.3–54.5) for RTOG 9911 versus 22.7% (95% CI, 13.4–33.5) for RTOG 9610 (Fig. 1A). These curves were generated in identical manners. In patients surviving >1 year, the cumulative incidence of subsequent late grade 4–5 toxicity was significantly higher than historical control (*P* = .02) with 5-year estimates of 22.4% (95% CI, 11.8–35.1) and 3.2% (95% CI, 0.2–14.5) for RTOG 9911 and RTOG 9610, respectively (Fig. 1B). We also analyzed cumulative grade 3–5 late toxicity using deaths versus deaths and progressions as competing events. As shown in Fig. E1, there was no substantial difference between the 2 analyses.

### Dose delivery

Treatment delivery has been previously reported. In brief, 73% of patients completed 4 cycles of chemotherapy, and chemotherapy was delivered per protocol or with minor variations in 81%. RT was delivered per protocol or with minor variations in 69%. Major deviations in RT occurred in 11%, while in 20%, RT was cut short by death (7%), disease progression (1%), or patient/clinician decision (12%).

### Survival and patterns of recurrence

**Fig. 2.**
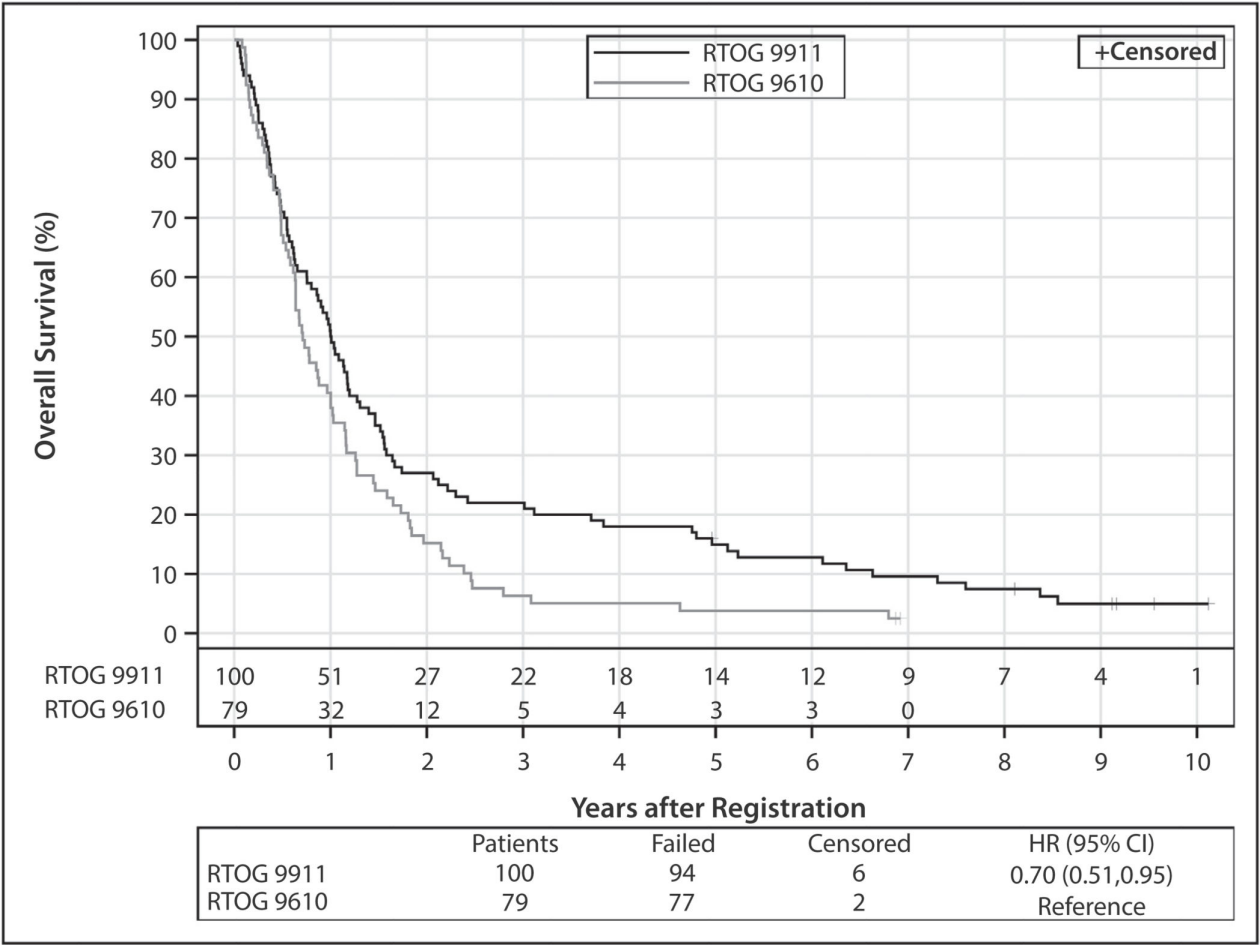
Kaplan-Meier estimates of overall survival compared with historical control, Radiation Therapy Oncology Group (RTOG) 9610. *Abbreviation:* HR = hazard ratio.

The median follow-up for surviving patients in this report is 9.1 years (range, 5.0–10.1 years) compared with 23.6 months for the original report. At this updated follow-up, 94 patients have died. OS was significantly improved over historical control (*P* = .01): median OS increased from 8.5 to 12 months, and 5-year OS rate from 3.8% (95% CI, 0.0–8.0) to 14.9% (95% CI, 7.9–21.9), corresponding to a hazard ratio of 0.70 (95% CI, 0.51–0.95) without adjustment for prognostic factors (Fig. 2, Table 3). After adjustment for Zubrod PS and prior surgery, 2 factors that were significantly associated with OS on multivariable analysis, the hazard ratio was 0.67 (95% CI, 0.49–0.92). There were fourteen 5-year survivors, of whom ≥1 was a 10-year survivor. Ninety-eight patients have had progressive disease or died; 5-year PFS is 7.0% (95% CI, 2.0–12.0). Seven patients were alive without progression of disease at 5 years, of whom 2 remained free of progression at 9 years. There were no significant differences in PFS and OS whether patients experienced recurrence of the original tumor or exhibited a new primary. For OS, the hazard ratio comparing new primary to recurrence was 1.57 (95% CI, 0.98–2.52) with *P* = .06 in univariate analysis and 1.32 (95% CI, 0.81–2.15) with *P* = .27 adjusted for Zubrod. For PFS, the hazard ratio comparing new primary to recurrence was 1.26 (95% CI, 0.79–2.02) with *P* = .33 in univariate analysis and 1.27 (95% CI, 0.75–2.14) with *P* = .38 adjusted for Zubrod and months from prior RT.

Patterns of failure and causes of death are summarized in Table 4: 64.9% have died from the incident cancer, 3.2% from SPT, and 22.3% from noncancer (11.7%) or unknown causes (10.6%). The causes of 11 noncancer deaths are listed in the footnote of Table 3; they included pulmonary etiology (n = 3), cardiac origin (n = 2), infection (n = 2), unknown illness (n = 1), suicide (n = 1), brainstem injury (n = 1), and carotid artery hemorrhage (n = 1). Because we do not have the attribution, it is unclear if the last 2 were related to treatment.

## Discussion

The median and 5-year survival rate for split course twice-daily fractionated RT with concurrent cisplatin and paclitaxel in locoregionally recurrent SCCHN proved promising and generally exceeded results observed with contemporaneous chemotherapy alone, the erstwhile standard in the setting of recurrent SCCHN, as well as prior attempts at chemo-reirradiation in our own group. In our initial analysis of this trial, survival was superior to our historic control (NRG Oncology RTOG 9610),^18^ which likewise featured split course twice-daily RT, albeit in conjunction with concurrent 5-fluorouracil and hydroxyurea. In the context of recurrent SCCHN, hydroxyurea is virtually never used outside of clinical trials; and 5-fluorouracil has also fallen out of favor, to a large extent, even though this agent in combination with platinum was the cytotoxic platform for the EXTREME study which documented a survival benefit favoring the addition of cetuximab in advanced, incurable disease.^19^ The survival benefit for 9911 over 9610^18^ has persisted over time with long-term follow-up, with no significant differences in OS or PFS observed between local recurrences and SPTs.

Despite improved survival compared with historic controls including those using chemotherapy alone, toxicity was substantial in this study, with a high incidence of both early and late grade 5 toxicities (9% total) and a 44% incidence of late grade ≥3 toxicity. This late toxicity rate was clearly higher for the regimen employed in RTOG 9911 compared with RTOG 9610. We believe this difference was partially an artifact of longer survival and therefore a higher percentage of patients at risk for late toxicity. We do not necessarily believe that this apparent increase had anything to do with the nature of the XRT employed; nor do we believe that the cisplatin/paclitaxel regimen was inherently more toxic than either fluorinated pyrimidines or hydroxyurea.

Overall, >20% of enrollees died of causes unrelated to the incident cancer, which, in addition to delayed toxicity, may have been due to an exacerbation of underlying comorbidities by study therapy. The toxicity rates observed in RTOG 9911 are in line with those reported in the randomized trial conducted by the Groupe d’Etude des Tumeurs de la Tête et du Cou and Groupe d’Oncologie Radiothérapie Tête Et Cou groups, where patients with resected locoregionally recurrent SCCHN were randomized to either observation or reirradiation with concurrent cisplatin chemotherapy.^20^ Of the reirradiated patients, 28% (17 of 60) experienced grade 3–4 acute toxicity, 39% (7 of 18) experienced grade 3–4 late toxicity at 2-years, and 8% (5 of 60 patients) died from treatment related causes (2 sepsis, 1 hemorrhage, 1 mucosal necrosis, and 1 severe laryngeal edema). Because of the higher rate of deaths related to treatment and secondary malignancy in the reirradiated arm, the OS was similar between the 2 arms, despite a significant improvement in locoregional control and disease-free survival, favoring reirradiation.

The reasons for apparently better outcomes in 9911 as compared with historical controls may be related to improved imaging, leading to earlier detection of locoregional recurrences and/or better selection of patients enrolled in this study. They may also be related to the addition of paclitaxel, which is an active agent in HNC. The addition of a taxane to cisplatin and 5-fluorouracil as induction chemotherapy has been shown to improve survival in patients with newly diagnosed locoregionally advanced SCCHN.^21–23^

Based on our initial analysis and the observation of 25% 2-year survival, we believed strongly at the time that a phase 3 trial directly comparing chemotherapy alone (arguably the previous standard approach in this setting) to concurrent chemotherapy and reirradiation was warranted with close attention to quality-adjusted survival. Unfortunately, the subsequent NRG Oncology RTOG trial which attempted to address this question was aborted due to slow accrual. Consequently, this question remains open.

Multiple additional limitations exist. Our data do not distinguish local recurrence from “marginal misses.” The location and pattern of failure were reported in the case report form from enrolling sites as local, regional or distant relapse. Local relapse did not distinguish between in-field versus marginal misses. Moreover, we did not have funding to collecting imaging at recurrence to support this type of analysis. Dosimetric details were not collected in the case report forms; hence we were unable to correlate dosimetry with late toxicities, including bone and vascular. T and N status as reported referred to the original diagnosis for patients with recurrence; we do not have “formal” rT and rN data. For SPT, we used staging at diagnosis. In addition, we relied on enrolling sites to determine whether the tumor was a recurrence or an SPT using the accepted definitions at the time, without specifying further defining criteria for each. Thus, there may have been some overlap between the 2 groups. Cross trial comparisons between studies are inherently “fraught,” but it should be noted that RTOG 9610 and RTOG 9911 employed virtually identical eligibility criteria with similar endpoints, they were conducted in the same cooperative group only a few years apart, and the investigators were almost all the same. Finally, we acknowledge that the data presented in this update are, to some extent, of historic interest. This study predates the use of proton beam, stereotactic body RT (SBRT), and immunotherapy and the routine use of IMRT, which, to a large extent, have rendered the regimen used in RTOG 9911 somewhat obsolete. On the other hand, RTOG 9911 establishes a threshold for outcomes against which newer regimens should be compared. In the MIRI collaborative retrospective series of reirradiation with IMRT for recurrent or secondary HNC, the local control rate at 2 years among 1635 patients was 52% and the OS rate was 46%, ostensibly better than observed in our trial. The pooled rates of late grade 3–5 toxicity were 26% with grade 5 toxicity rate of 3.1%.

How best to pursue reirradiation in SCCHN in the current era remains an open question. In the recent therapeutic era just preceding the contemporary age of immunotherapy, most patients who presented with newly diagnosed, unresectable locally advanced HNC received combined modality therapy including either cisplatin or cetuximab,^24–33^ whereas only 20% of those enrolled in our trial were exposed to prior chemotherapy. In this regard, since so many patients with locoregional recurrence in the modern era are likely to have been exposed to prior platinum, it is important to test alternative radio-sensitizers, novel targeted drugs, or to alter the nature of the RT so tumor exposure is maximized and the collateral damage to adjacent vital structures is minimized.^34–36^ Since 2018, immunotherapy has become a standard approach in patients with SCCHN with recurrent or metastatic disease. Pembrolizumab and other checkpoint inhibitors, either alone or in combination with chemotherapy, have emerged as front-line therapy in this setting, with most studies identifying a small percentage of long-term survivors.^37–41^ How to integrate checkpoint inhibitors into the reirradiation paradigm is an ongoing question. For example, Wong and colleagues have evaluated SBRT in combination with both concurrent and adjuvant pembrolizumab in patients with locoregionally recurrent SCCHN and have demonstrated safety^42^; the contribution of pembrolizumab after reirradiation with SBRT is now being tested in a phase 2 randomized trial (RTOGF 3507). Investigators have also tested the role of nivolumab in combination with IMRT in the same setting.^43^

In addition, there remains significant concern about interrupting the radiation course, which in the treatment-naïve setting has been associated with impaired survival.^44–46^ The use of small field SBRT has been tested in patients with locoregionally recurrent SCCHN in phase 1–2 trials. Early reports suggested excellent local control, good quality of life, and promising 1-year survival, especially in patients with smaller tumors.^47–49^ However, the MIRI series indicated inferiority of SBRT to conventional reirradiation especially for larger-size tumors.^50^ Furthermore, robust data on long-term toxicity is lacking with this approach, and without these, this treatment should still be considered investigational.

Even with advances in RT delivery, many clinicians are wary of reirradiation because of the specter of toxicity and the risk of grade 5 events, including vascular hemorrhage. The incidence of carotid rupture documented in our study and others underscores this concern.^41,51^ Proximity to vital vascular structures and the size of the RT field may prove to be important discriminants both with respect to success and toxicity.^52^ Appropriate patient selection restricting this approach to patients with excellent PS should help mitigate reservations. In addition, adequate nutrition is imperative; those with compromised nutritional integrity will likely need enteral feedings via nasogastric or gastrostomy tubes.

Newer studies integrating immunotherapy with chemotherapy and reirradiation or comparing immunotherapy alone to reirradiation and chemotherapy will be informative. To this end, EA3191 randomizes patients after resection of recurrent SCCHN to either pembrolizumab alone administered every 6 weeks for 9 cycles versus IMRT or proton beam XRT (30 fractions) given concurrently with either carboplatin or cisplatin.

In summary, reirradiation in locally recurrent or second primary HNC, in the absence of distant metastases, remains a viable option for treatment and potential long-term survival. Optimization with modern systemic therapies and/or reirradiation techniques may further improve these outcomes.

## Supplementary Material

2

Supplementary material associated with this article can be found, in the online version, at doi:10.1016/j.ijrobp.2025.07.1434.

## Figures and Tables

**Table 1:** Patient and tumor characteristics prior to reirradiation (n = 100)

Characteristic	# (%)

Age (y)	
Median	60
Min-max	27–83
Sex	
Male	76 (76.0%)
Female	24 (24.0%)
Race	
White	90 (90.0%)
Black or African-American	10 (10.0%)
Zubrod performance status	
0	34 (34.0%)
1	66 (66.0%)
Feeding tube	
No	53 (53.0%)
Yes	47 (47.0%)
Type of disease at study entry	
Recurrence of original head and neck tumor	77 (77.0%)
New head and neck primary tumor	23 (23.0%)
Location of disease at study entry	
Local only	58 (58.0%)
Regional only	12 (12.0%)
Local and regional	30 (30.0%)
T stage*	
T1	15 (15.0%)
T2	35 (35.0%)
T3	15 (15.0%)
T4	30 (30.0%)
TIS	1 (1.0%)
Tx	1 (1.0%)
Unknown	3 (3.0%)
N stage*	
N0	51 (51.0%)
N1	21 (21.0%)
N2a	4 (4.0%)
N2b	13 (13.0%)
N2c	5 (5.0%)
N3	4 (4.0%)
Unknown	2 (2.0%)
Primary site at study entry	
Oral cavity	27 (27.0%)
Oropharynx	41 (41.0%)
Hypopharynx	12 (12.0%)
Larynx	10 (10.0%)
Other	10 (10.0%)
Months from prior radiation therapy to head and neck	
Median	39.6
Min-max	6.1–317.9
Months from prior radiation therapy to head and neck (recurrence of original head and neck tumor)	(n = 77)
Median	28.6
Min-max	6.1–216.2
Months from prior radiation therapy to head and neck (new head and neck primary tumor)	(n = 23)
Median	85.0
Min-max	17.4–317.9
Prior radiation therapy dose to head and neck (Gy)	
Median	65.7
Min-max	45.0–75.0
Prior chemotherapy	
No	80 (80.0%)
Yes	20 (20.0%)
Prior chemotherapy agent(s)	(n = 20)
CCNU	1 (5.0%)
5-FU	3 (15.0%)
Platinum	2 (10.0%)
Procarbazine	1 (5.0%)
Carboplatin	2 (10.0%)
Multiple	9 (45.0%)
Unknown	2 (10.0%)
Prior surgery for head and neck cancer	
No	23 (23.0%)
Yes	77 (77.0%)

* For recurrences, staging is from original cancer. For second primaries, staging is from study cancer.

**Table 2:** Late radiation therapy toxicity (n = 86)

Organ System	Grade				
	1	2	3	4	5

Worst grade overall	9 (10.5%)	17 (19.8%)	13 (15.1%)	15 (17.4%)	4 (4.7%)
Bone	4 (4.7%)	0	0	8 (9.3%)	0
Esophagus/pharynx	7 (8.1%)	13 (15.1%)	14 (16.3%)	3 (3.5%)	0
Joint	4 (4.7%)	6 (7.0%)	3 (3.5%)	0	0
Larynx	18 (20.9%)	4 (4.7%)	1 (1.2%)	1 (1.2%)	0
Mucous membrane	17 (19.8%)	13 (15.1%)	1 (1.2%)	6 (7.0%)	0
Salivary gland	15 (17.4%)	18 (20.9%)	7 (8.1%)	0	0
Skin	18 (20.9%)	14 (16.3%)	1 (1.2%)	2 (2.3%)	0
Spinal cord	2 (2.3%)	0	0	0	0
Subcutaneous tissue	10 (11.6%)	18 (20.9%)	5 (5.8%)	5 (5.8%)	1 (1.2%)
Other	6 (7.0%)	6 (7.0%)	5 (5.8%)	4 (4.7%)	3* (3.5%)

* Carotid artery rupture—2; hemorrhage—1.

**Table 3:** Overall survival for RTOG 9911 compared with historical control, RTOG 9610

	RTOG 9911				RTOG 9610			
Year	Estimate (%)	95% CI (%)	Cumulative deaths	At risk	Estimate (%)	95% CI (%)	Cumulative deaths	At risk

0	100.0	−	0	100	100.0	−	0	79
1	50.0	40.2–59.8	50	51	40.5	29.7–51.3	47	32
2	27.0	18.3–35.7	73	27	15.2	7.3–23.1	67	12
3	22.0	13.9–30.1	78	22	6.3	1.0–11.7	74	5
4	18.0	10.5–25.5	82	18	5.1	0.2–9.9	75	4
5	14.9	7.9–21.9	85	14	3.8	0.0–8.0	76	3
6	12.8	6.2–19.4	87	12	3.8	0.0–8.0	76	3
7	9.6	3.7–15.5	90	9				
8	7.5	2.2–12.7	92	7				
9	5.0	0.5–9.5	94	4				
10	5.0	0.5–9.5	94	1				
Total			94				77	

*Abbreviation:* RTOG = Radiation Therapy Oncology Group.

Median survival (95% CI).

RTOG 9911: 12.0 months (9.0–14.4).

RTOG 9610: 8.5 months (7.4–12.0).

One-sided log-rank test: *P* = .01.

Hazard ratio (RTOG 9911/RTOG 9610) (95% CI).

Univariate: 0.70 (0.51–0.95).

Multivariable: 0.67 (0.49–0.92) (adjusted for Zubrod performance status and prior surgery).

**Table 4:** Patterns of failure and causes of death

First failure (progression-free survival)	(n = 98)

Local progression	22 (22.4%)
Regional progression	10 (10.2%)
Local and regional progression	1 (1.0%)
Local progression and distant metastases	1 (1.0%)
Local progression and second primary	1 (1.0%)
Distant metastases	10 (10.2%)
Second primary	9 (9.2%)
Dead-study cancer NOS	24 (24.5%)
Dead-no progression	20 (20.4%)
Cause of death	(n = 94)
Study cancer	61 (64.9%)
Second primary	3 (3.2%)
Protocol treatment	9 (9.6%)
Other*	11 (11.7%)
Unknown	10 (10.6%)

*Abbreviation:* NOS = not otherwise specified.

* Respiratory failure—2; arrhythmia—1; brain stem injury—1; carotid artery hemorrhage—1; chronic obstructive pulmonary disease—1; intercurrent illness NOS—1; myocardial infarction—1; sepsis—1; sepsis, pneumonia, cerebral vascular accident—1; suicide—1.

## Data Availability

All data will be made available per the NCTN Data Archive rules. The link for the archive is: https://nctn-data-archive.nci.nih.gov/.
