# Supplementary material for: Phase 2 Study of Low-Dose Paclitaxel and Cisplatin in Combination With Split-Course Concomitant Twice-Daily Reirradiation in Recurrent Squamous Cell Carcinoma of the Head and Neck: Long-term Follow-up of NRG Oncology Radiation Therapy Oncology Group Protocol 9911
Source: Int J Radiat Oncol Biol Phys. Author manuscript; Available in PMC 2026 Jan 12. (PMC12790980; doi:10.1016/j.ijrobp.2025.07.1434)
Supplement: 2 [file NIHMS2110591-supplement-2.docx]

| **Supplemental Table 1 Chemotherapy and Acute Radiotherapy Toxicity (n=100)** | | | | | |
| --- | --- | --- | --- | --- | --- |
| Category | Grade | | | | |
| Term | 1 | 2 | 3 | 4 | 5 |
|  | | | | | |
| Worst grade – non-hematologic | 6  (6.0%) | 22  (22.0%) | 53  (53.0%) | 13  (13.0%) | 4  (4.0%) |
|  |  |  |  |  |  |
| Worst grade -- overall | 2  (2.0%) | 17  (17.0%) | 51  (51.0%) | 23  (23.0%) | 5  (5.0%) |
|  |  |  |  |  |  |
| ALLERGY/IMMUNOLOGY | 0 | 1  (1.0%) | 0 | 0 | 0 |
| Allergy-Other | 0 | 1  (1.0%) | 0 | 0 | 0 |
|  | | | | | |
| AUDITORY/HEARING | 3  (3.0%) | 9  (9.0%) | 2  (2.0%) | 0 | 0 |
| Hearing impaired | 0 | 6  (6.0%) | 2  (2.0%) | 0 | 0 |
| Hearing-Other | 3  (3.0%) | 4  (4.0%) | 0 | 0 | 0 |
| Otitis media serous NOS | 0 | 1  (1.0%) | 0 | 0 | 0 |
|  | | | | | |
| BLOOD/BONE MARROW | 14  (14.0%) | 35  (35.0%) | 24  (24.0%) | 20  (20.0%) | 1  (1.0%) |
| Hemoglobin decreased | 21  (21.0%) | 52  (52.0%) | 19  (19.0%) | 2  (2.0%) | 0 |
| Leukopenia NOS | 23  (23.0%) | 14  (14.0%) | 11  (11.0%) | 19  (19.0%) | 0 |
| Lymphopenia | 1  (1.0%) | 4  (4.0%) | 5  (5.0%) | 0 | 0 |
| Neutropenia | 15  (15.0%) | 7  (7.0%) | 6  (6.0%) | 17  (17.0%) | 1  (1.0%) |
| Packed red blood cell transfusion | 0 | 0 | 11  (11.0%) | 0 | 0 |
| Platelet count decreased | 26  (26.0%) | 6  (6.0%) | 5  (5.0%) | 1  (1.0%) | 0 |
| Platelet transfusion | 0 | 0 | 1  (1.0%) | 0 | 0 |
|  | | | | | |
| CARDIOVASCULAR (ARRHYTHMIA) | 4  (4.0%) | 0 | 2  (2.0%) | 0 | 0 |
| Arrhythmia NOS | 0 | 0 | 1  (1.0%) | 0 | 0 |
| Electrocardiogram QT prolonged | 1  (1.0%) | 0 | 0 | 0 | 0 |
| Nodal arrhythmia | 1  (1.0%) | 0 | 0 | 0 | 0 |
| Sinus tachycardia | 2  (2.0%) | 0 | 0 | 0 | 0 |
| Supraventricular arrhythmia NOS | 0 | 0 | 2  (2.0%) | 0 | 0 |
| Ventricular arrhythmia NOS | 0 | 0 | 1  (1.0%) | 0 | 0 |
|  | | | | | |
| CARDIOVASCULAR (GENERAL) | 6  (6.0%) | 9  (9.0%) | 6  (6.0%) | 0 | 0 |
| Circulatory or cardiac-Other | 0 | 1  (1.0%) | 0 | 0 | 0 |
| Edema NOS | 7  (7.0%) | 2  (2.0%) | 1  (1.0%) | 0 | 0 |
| Hypertension NOS | 2  (2.0%) | 0 | 1  (1.0%) | 0 | 0 |
| Hypotension NOS | 1  (1.0%) | 7  (7.0%) | 3  (3.0%) | 0 | 0 |
| Phlebitis superficial | 0 | 1  (1.0%) | 0 | 0 | 0 |
| Thrombosis NOS | 0 | 0 | 2  (2.0%) | 0 | 0 |
|  | | | | | |
| COAGULATION | 1  (1.0%) | 0 | 0 | 0 | 0 |
| Activated partial thromboplastin time prolonged | 1  (1.0%) | 0 | 0 | 0 | 0 |
| Prothrombin time prolonged | 1  (1.0%) | 0 | 0 | 0 | 0 |
|  | | | | | |
| CONSTITUTIONAL SYMPTOMS | 16  (16.0%) | 40  (40.0%) | 12  (12.0%) | 0 | 0 |
| Constitutional symptoms-Other | 0 | 0 | 1  (1.0%) | 0 | 0 |
| Fatigue | 17  (17.0%) | 31  (31.0%) | 9  (9.0%) | 0 | 0 |
| Pyrexia | 7  (7.0%) | 4  (4.0%) | 0 | 0 | 0 |
| Rigors | 7  (7.0%) | 0 | 1  (1.0%) | 0 | 0 |
| Sweating increased | 0 | 1  (1.0%) | 0 | 0 | 0 |
| Weight decreased | 9  (9.0%) | 21  (21.0%) | 2  (2.0%) | 0 | 0 |
|  | | | | | |
| DERMATOLOGY/SKIN | 30  (30.0%) | 43  (43.0%) | 9  (9.0%) | 1  (1.0%) | 0 |
| Alopecia | 13  (13.0%) | 20  (20.0%) | 0 | 0 | 0 |
| Culture wound positive | 0 | 2  (2.0%) | 0 | 0 | 0 |
| Dermatitis exfoliative NOS | 3  (3.0%) | 1  (1.0%) | 0 | 0 | 0 |
| Dermatitis radiation NOS | 46  (46.0%) | 27  (27.0%) | 8  (8.0%) | 0 | 0 |
| Dry skin | 2  (2.0%) | 0 | 0 | 0 | 0 |
| Erythema multiforme | 2  (2.0%) | 0 | 2  (2.0%) | 0 | 0 |
| Injection site reaction NOS | 0 | 1  (1.0%) | 0 | 0 | 0 |
| Photosensitivity reaction NOS | 1  (1.0%) | 0 | 0 | 0 | 0 |
| Pruritus NOS | 1  (1.0%) | 0 | 0 | 0 | 0 |
| Skin discoloration | 5  (5.0%) | 0 | 0 | 0 | 0 |
| Skin-Other | 5  (5.0%) | 2  (2.0%) | 0 | 0 | 0 |
| Toxicoderma | 0 | 1  (1.0%) | 0 | 0 | 0 |
| Urticaria NOS | 2  (2.0%) | 0 | 0 | 1  (1.0%) | 0 |
|  | | | | | |
| ENDOCRINE | 0 | 4  (4.0%) | 0 | 0 | 0 |
| Endocrine-Other | 0 | 1  (1.0%) | 0 | 0 | 0 |
| Hypothyroidism | 0 | 3  (3.0%) | 0 | 0 | 0 |
|  | | | | | |
| GASTROINTESTINAL | 8  (8.0%) | 33  (33.0%) | 46  (46.0%) | 4  (4.0%) | 1  (1.0%) |
| Anorexia | 5 (5.0%) | 9  (9.0%) | 5  (5.0%) | 3  (3.0%) | 0 |
| Constipation | 6  (6.0%) | 7  (7.0%) | 0 | 0 | 0 |
| Dehydration | 0 | 7  (7.0%) | 15  (15.0%) | 0 | 1  (1.0%) |
| Diarrhea (with colostomy) | 1  (1.0%) | 1  (1.0%) | 1  (1.0%) | 0 | 0 |
| Diarrhea NOS | 18  (18.0%) | 6  (6.0%) | 5  (5.0%) | 0 | 0 |
| Dry mouth | 6  (6.0%) | 8  (8.0%) | 0 | 0 | 0 |
| Duodenal ulcer | 0 | 0 | 1  (1.0%) | 0 | 0 |
| Dyspepsia | 3  (3.0%) | 1  (1.0%) | 0 | 0 | 0 |
| Dysphagia | 13  (13.0%) | 22  (22.0%) | 20  (20.0%) | 2  (2.0%) | 0 |
| Esophageal spasm | 1  (1.0%) | 2  (2.0%) | 0 | 0 | 0 |
| Esophagitis NOS | 3  (3.0%) | 6  (6.0%) | 4  (4.0%) | 0 | 0 |
| Fistula-pharyngeal | 0 | 0 | 2  (2.0%) | 0 | 0 |
| Flatulence | 1  (1.0%) | 0 | 0 | 0 | 0 |
| GI-other | 0 | 2  (2.0%) | 0 | 0 | 0 |
| Gastric ulcer | 0 | 0 | 1  (1.0%) | 0 | 0 |
| Ileus | 0 | 1  (1.0%) | 0 | 0 | 0 |
| Nausea | 38  (38.0%) | 17  (17.0%) | 19  (19.0%) | 0 | 0 |
| Radiation mucositis | 29  (29.0%) | 30  (30.0%) | 8  (8.0%) | 1  (1.0%) | 0 |
| Salivary gland disorder NOS | 24  (24.0%) | 25  (25.0%) | 2  (2.0%) | 0 | 0 |
| Stomatitis | 19  (19.0%) | 26  (26.0%) | 6  (6.0%) | 1  (1.0%) | 0 |
| Stomatitis/pharyngitis for BMT | 0 | 0 | 1  (1.0%) | 0 | 0 |
| Taste disturbance | 5  (5.0%) | 9  (9.0%) | 0 | 0 | 0 |
| Tracheo-oesophageal fistula NOS | 0 | 0 | 1  (1.0%) | 0 | 0 |
| Vomiting NOS | 19  (19.0%) | 21  (21.0%) | 14  (14.0%) | 0 | 0 |
|  | | | | | |
| HEMORRHAGE | 3  (3.0%) | 1  (1.0%) | 4  (4.0%) | 1  (1.0%) | 0 |
| Epistaxis | 1  (1.0%) | 0 | 0 | 0 | 0 |
| Hematemesis | 1  (1.0%) | 0 | 1  (1.0%) | 0 | 0 |
| Hematuria present | 0 | 1  (1.0%) | 0 | 0 | 0 |
| Hemoptysis | 2  (2.0%) | 0 | 0 | 0 | 0 |
| Hemorrhage NOS | 0 | 0 | 2  (2.0%) | 1  (1.0%) | 0 |
| Hemorrhage-Other | 0 | 0 | 1  (1.0%) | 0 | 0 |
| Hemorrhagic stroke | 0 | 0 | 1  (1.0%) | 0 | 0 |
|  | | | | | |
| HEPATIC | 22  (22.0%) | 20  (20.0%) | 5  (5.0%) | 0 | 0 |
| Alanine aminotransferase increased | 5  (5.0%) | 0 | 0 | 0 | 0 |
| Aspartate aminotransferase increased | 9  (9.0%) | 0 | 0 | 0 | 0 |
| Blood albumin decreased | 11  (11.0%) | 17  (17.0%) | 5  (5.0%) | 0 | 0 |
| Blood alkaline phosphatase NOS increased | 21  (21.0%) | 1  (1.0%) | 0 | 0 | 0 |
| Blood bilirubin increased | 3  (3.0%) | 3  (3.0%) | 0 | 0 | 0 |
| Gamma-glutamyltransferase increased | 1  (1.0%) | 0 | 0 | 0 | 0 |
| Hepatic failure | 1  (1.0%) | 0 | 0 | 0 | 0 |
| Hepatic-Other | 1  (1.0%) | 0 | 0 | 0 | 0 |
|  | | | | | |
| INFECTION FEBRILE NEUTROPENIA | 2  (2.0%) | 12  (12.0%) | 11  (11.0%) | 3  (3.0%) | 1  (1.0%) |
| Febrile neutropenia | 0 | 1  (1.0%) | 3  (3.0%) | 2  (2.0%) | 0 |
| Implant infection | 0 | 2  (2.0%) | 0 | 0 | 0 |
| Infection NOS | 1  (1.0%) | 8  (8.0%) | 5  (5.0%) | 0 | 0 |
| Infection with grade 3 or 4 neutropenia | 0 | 0 | 3  (3.0%) | 1  (1.0%) | 1  (1.0%) |
| Infection with unknown ANC | 0 | 0 | 1  (1.0%) | 0 | 0 |
| Infection, Other | 1  (1.0%) | 1  (1.0%) | 0 | 0 | 0 |
|  | | | | | |
| LYMPHATICS | 1  (1.0%) | 2  (2.0%) | 0 | 0 | 0 |
| Lymphangiopathy NOS | 1  (1.0%) | 0 | 0 | 0 | 0 |
| Lymphatics-Other | 0 | 2  (2.0%) | 0 | 0 | 0 |
|  | | | | | |
| METABOLIC/LABORATORY | 23  (23.0%) | 15  (15.0%) | 23  (23.0%) | 9  (9.0%) | 0 |
| Alkalosis NOS | 1  (1.0%) | 0 | 0 | 0 | 0 |
| Blood bicarbonate decreased | 2  (2.0%) | 0 | 0 | 0 | 0 |
| Blood creatinine phosphokinase increased | 1  (1.0%) | 0 | 0 | 0 | 0 |
| Blood magnesium decreased | 14  (14.0%) | 8  (8.0%) | 5  (5.0%) | 6  (6.0%) | 0 |
| Hypercalcemia | 2  (2.0%) | 1  (1.0%) | 3  (3.0%) | 0 | 0 |
| Hyperglycemia NOS | 20  (20.0%) | 9  (9.0%) | 2  (2.0%) | 0 | 0 |
| Hyperkalemia | 10  (10.0%) | 6  (6.0%) | 0 | 0 | 0 |
| Hypermagnesemia | 7  (7.0%) | 1  (1.0%) | 1  (1.0%) | 0 | 0 |
| Hypernatremia | 3  (3.0%) | 0 | 0 | 0 | 0 |
| Hyperuricemia | 3  (3.0%) | 1  (1.0%) | 0 | 0 | 0 |
| Hypocalcemia | 11  (11.0%) | 13  (13.0%) | 9  (9.0%) | 5  (5.0%) | 0 |
| Hypoglycaemia NOS | 4  (4.0%) | 0 | 0 | 0 | 0 |
| Hypokalemia | 11  (11.0%) | 1  (1.0%) | 13  (13.0%) | 0 | 0 |
| Hyponatremia | 32  (32.0%) | 1  (1.0%) | 15  (15.0%) | 1  (1.0%) | 0 |
| Hypophosphatemia | 5  (5.0%) | 2  (2.0%) | 5  (5.0%) | 0 | 0 |
| Metabolic-Other | 2  (2.0%) | 2  (2.0%) | 0 | 0 | 0 |
|  | | | | | |
| MUSCULOSKELETAL | 4  (4.0%) | 6  (6.0%) | 0 | 0 | 0 |
| Joint, muscle, or bone-Other | 4  (4.0%) | 0 | 0 | 0 | 0 |
| Muscle weakness NOS | 1  (1.0%) | 6  (6.0%) | 0 | 0 | 0 |
|  | | | | | |
| NEUROLOGY | 13  (13.0%) | 5  (5.0%) | 3  (3.0%) | 0 | 1  (1.0%) |
| Anxiety NEC | 0 | 2  (2.0%) | 0 | 0 | 0 |
| Cerebral ischaemia | 0 | 0 | 0 | 0 | 1  (1.0%) |
| Cranial nerve injury NOS | 1  (1.0%) | 0 | 0 | 0 | 0 |
| Dizziness (exc vertigo) | 6  (6.0%) | 3  (3.0%) | 0 | 0 | 0 |
| Insomnia NEC | 4  (4.0%) | 1  (1.0%) | 0 | 0 | 0 |
| Peripheral motor neuropathy | 0 | 0 | 1  (1.0%) | 0 | 0 |
| Peripheral sensory neuropathy | 8  (8.0%) | 1  (1.0%) | 1  (1.0%) | 0 | 0 |
| Syncope | 0 | 0 | 1  (1.0%) | 0 | 0 |
| Vertigo NEC | 0 | 1  (1.0%) | 0 | 0 | 0 |
|  | | | | | |
| OCULAR/VISUAL | 0 | 1  (1.0%) | 0 | 0 | 0 |
| Vision blurred | 0 | 1  (1.0%) | 0 | 0 | 0 |
|  | | | | | |
| PAIN | 17  (17.0%) | 19  (19.0%) | 6  (6.0%) | 0 | 0 |
| Abdominal pain NOS | 1  (1.0%) | 2  (2.0%) | 0 | 0 | 0 |
| Arthralgia | 8  (8.0%) | 2  (2.0%) | 0 | 0 | 0 |
| Bone pain | 2  (2.0%) | 0 | 0 | 0 | 0 |
| Chest pain | 0 | 1  (1.0%) | 0 | 0 | 0 |
| Earache | 1  (1.0%) | 0 | 0 | 0 | 0 |
| Headache NOS | 4  (4.0%) | 0 | 1  (1.0%) | 0 | 0 |
| Myalgia | 4  (4.0%) | 2  (2.0%) | 1  (1.0%) | 0 | 0 |
| Pain due to radiation | 2  (2.0%) | 15  (15.0%) | 3  (3.0%) | 0 | 0 |
| Pain-other | 3  (3.0%) | 2  (2.0%) | 1  (1.0%) | 0 | 0 |
|  | | | | | |
| PULMONARY | 15  (15.0%) | 15  (15.0%) | 6  (6.0%) | 0 | 2  (2.0%) |
| Cough | 5  (5.0%) | 3  (3.0%) | 1  (1.0%) | 0 | 0 |
| Dysphonia | 12  (12.0%) | 11  (11.0%) | 2  (2.0%) | 0 | 0 |
| Dyspnea NOS | 1  (1.0%) | 2  (2.0%) | 0 | 0 | 0 |
| Hiccups | 2  (2.0%) | 0 | 1  (1.0%) | 0 | 0 |
| Hypoxia | 0 | 0 | 2  (2.0%) | 0 | 0 |
| Pleural effusion | 1  (1.0%) | 0 | 0 | 0 | 0 |
| Pneumonitis NOS | 1  (1.0%) | 0 | 3  (3.0%) | 0 | 2  (2.0%) |
| Pneumothorax NOS | 0 | 1  (1.0%) | 0 | 0 | 0 |
| Pulmonary-other | 0 | 3  (3.0%) | 0 | 0 | 0 |
|  | | | | | |
| RENAL/GENITOURINARY | 14  (14.0%) | 3  (3.0%) | 6  (6.0%) | 0 | 0 |
| Blood creatinine increased | 10  (10.0%) | 4  (4.0%) | 4  (4.0%) | 0 | 0 |
| Fanconi syndrome | 0 | 0 | 1  (1.0%) | 0 | 0 |
| Hemoglobinuria present | 1  (1.0%) | 0 | 0 | 0 | 0 |
| Renal failure NOS | 0 | 0 | 1  (1.0%) | 0 | 0 |
| Renal/GU-Other | 3  (3.0%) | 1  (1.0%) | 0 | 0 | 0 |
| Urogenital fistula | 0 | 0 | 1  (1.0%) | 0 | 0 |
|  | | | | | |
| UNKNOWN | 3  (3.0%) | 4  (4.0%) | 0 | 0 | 0 |
|  | | | | | |
| Toxicities were graded with Common Toxicity Criteria version 2.0. | | | | | |

**Supplemental Figure 1: Cummulative Grade 3-5 late toxicity analyzed with deaths vs. deaths and progressions as completing events.**

**
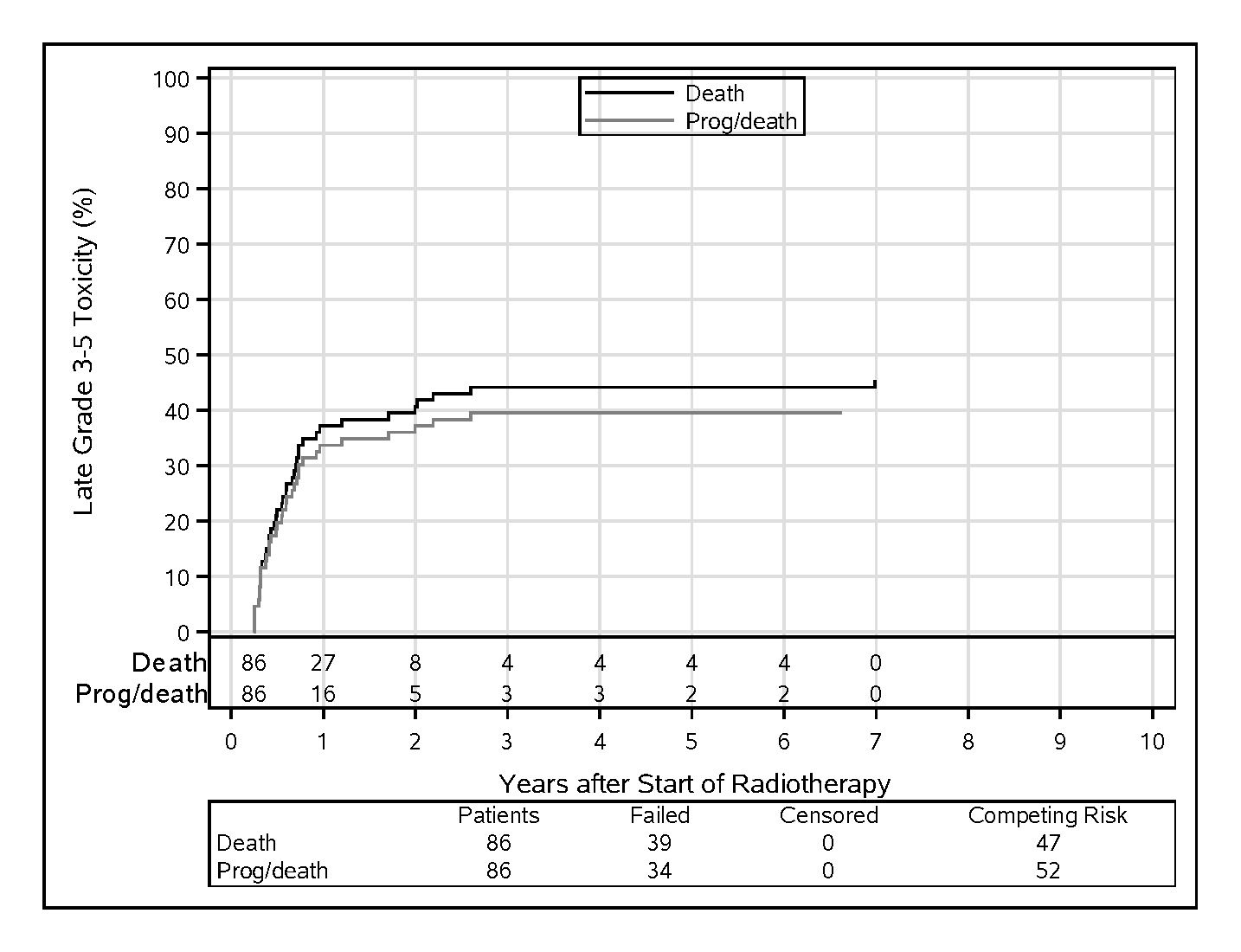
**
